# A Rapid and Reliable qPCR Method for Genetic Purity Testing of BT-Type CMS Rice Seed Lots

**DOI:** 10.3390/cimb48060576

**Published:** 2026-06-01

**Authors:** Bilian Hu, Yuting Dai, Can Cheng, Jihua Zhou, Fuan Niu, Bin Sun, Anpeng Zhang, Liming Cao, Huangwei Chu

**Affiliations:** 1 College of Fisheries and Life Science, Shanghai Ocean University, Shanghai 201306, China; bilianaya@163.com; 2 Key Laboratory of Germplasm Innovation and Genetic Improvement of Grain and Oil Crops (Co-Construction by Ministry and Province), Ministry of Agriculture and Rural Affairs, Crop Breeding and Cultivation Research Institute, Shanghai Academy of Agricultural Sciences, Shanghai 201403, China; daiyuting@saas.sh.cn (Y.D.); chengcan@saas.sh.cn (C.C.); zhoujihua@saas.sh.cn (J.Z.); niufuan@saas.sh.cn (F.N.); sunbin@saas.sh.cn (B.S.); zhanganpeng@saas.sh.cn (A.Z.)

**Keywords:** rice, *japonica* hybrid rice, BT-type cytoplasmic male sterility (CMS), seed genetic purity, quantitative real-time PCR (qPCR)

## Abstract

Boro II (BT), the first cytoplasmic male sterility (CMS) system in rice, is widely used in three-line *japonica* hybrid rice production. Accurate detection of maintainer-seed contamination in BT-type CMS seed lots is critical for ensuring genetic purity and hybrid seed quality. In this study, we developed a SYBR Green-based quantitative real-time PCR (qPCR) assay for the detection and quantification of maintainer-seed contamination in BT-type CMS seed lots. Maintainer-specific primers targeting a mitochondrial sequence unique to the maintainer line, together with an endogenous reference targeting a conserved mitochondrial sequence present in both maintainer and CMS lines, were validated for specificity. A standard curve was constructed using defined CMS–maintainer seed mixtures (0.1–5% contamination), and ΔCt values were converted to relative abundance (2^−ΔCt^). The assay exhibited high specificity, reproducibility, and sensitivity, with a strong linear relationship between 2^−ΔCt^ values and actual contamination levels (*R*^2^ > 0.99). Performance testing using simulated contamination samples (0.2–3.13%) demonstrated accurate quantification with acceptable recovery rates. This method provides a rapid, robust, and reliable tool for routine genetic purity testing and quality control in BT-type CMS hybrid rice seed production.

## 1. Introduction

Hybrid rice generally exhibits a 20–30% yield advantage over conventional inbred rice varieties, and its commercialization has substantially increased both yield per unit area and total rice production [1]. High seed purity is essential for sustaining the productivity and development of hybrid rice, and hybrid seed production, therefore, requires strict quality control. According to the current Chinese national standard (GB 4404.1-2024) [2], hybrid rice seeds supplied to farmers must meet a minimum purity requirement of 97%, whereas the parental lines used in three-line hybrid rice production, including the male sterile line, maintainer line, and restorer line, are required to reach a higher purity standard of 99.5%. Based on our production experience, the major off-type plants in *japonica* hybrid rice are cytoplasmic male sterile (CMS) plants and their corresponding maintainer plants, a pattern consistent with observations in indica hybrid rice [3,4]. These off-type plants primarily arise from contamination of the CMS population by its corresponding maintainer plants during the F_1_ seed production process [5]. Therefore, ensuring the genetic purity of CMS seed is a key prerequisite for ensuring the purity of hybrid seeds.

Conventionally, the genetic purity of CMS seed has been assessed using the grow-out test (GOT) [6]. To meet the stringent purity requirement of ≥99.5% for CMS lines, at least 800 plants must be evaluated in each GOT plot (Chinese national standard GB/T 3543.5-2025) [7]. However, due to the shared isonuclear background between CMS lines and their corresponding maintainer lines, it is extremely difficult to distinguish the two before flowering, which limits the suitability of GOT for rapid or large-scale CMS seed purity assessment [3,6]. Therefore, developing and applying genetically informative approaches to evaluate CMS line purity would substantially improve the detection of seed lots contaminated with maintainer lines.

Compared with GOT, molecular marker-based approaches for evaluating CMS purity offer several advantages, including faster processing and higher reproducibility [3,6,8,9]. However, these methods are generally qualitative, and assessing the purity of a batch of CMS seeds often requires DNA extraction from hundreds of individual seeds, followed by PCR amplification and gel electrophoresis. Consequently, when the genetic purity of a large number of samples needs to be tested, the process becomes laborious and time-consuming. To address these limitations, Anupama et al. (2020) developed a method for testing the purity of bulked parental line (A-line) seeds using capillary electrophoresis [6].

In previously developed qualitative PCR-based approaches for CMS purity assessment, co-dominant markers capable of differentiating CMS lines from their corresponding maintainer lines [8,10,11], or CMS-specific markers [3,4,9], were used to amplify DNA extracted from individual seeds. Through this single-seed genotyping strategy, each seed could be classified as either a true CMS seed or a contaminating maintainer seed. Quantitative real-time PCR (qPCR) is a robust and sensitive analytical platform widely used for quantitative detection in various fields [12,13]; however, quantitative qPCR-based approaches for bulk-level assessment of CMS seed purity remain insufficiently explored. In contrast to previously reported qualitative PCR-based approaches, establishment of a quantitative detection system for CMS seed purity requires the identification of a maintainer-line-specific target suitable for relative quantification. In the present study, a maintainer-specific mitochondrial sequence was selected as the quantitative target, while a conserved mitochondrial sequence shared by both CMS and maintainer lines was used as the endogenous reference. By measuring the abundance of the maintainer-specific target relative to the endogenous reference, the proportion of maintainer-seed contamination in bulk CMS seed lots can be quantitatively estimated. Because CMS lines and their corresponding maintainer lines share identical nuclear genetic backgrounds, mitochondrial genomic polymorphisms provide an effective source for target development. The strategy established here was designed for practical purity assessment of BT-type CMS rice currently used in hybrid rice production and may provide a transferable framework for quantitative purity testing of other CMS systems following system-specific target validation and calibration.

Boro II (BT), the first CMS system identified in rice and originally derived from the cytoplasm of the indica variety Chinsurah Boro II [14], has been mainly used in the production of three-line *japonica* hybrid rice. In this study, a novel SYBR Green-based qPCR assay was developed for detecting and quantifying maintainer-seed contamination in BT-type CMS seed lots. The results demonstrated a strong linear relationship between relative qPCR signals and maintainer-seed contamination levels, enabling accurate quantitative assessment. Thus, the developed assay provides a robust and practical tool for routine genetic purity testing in BT-type CMS rice seed production.

## 2. Materials and Methods

### 2.1. Rice Materials

Seeds of BT-type CMS lines (Shenchang A, Shen 21A, Shen 22A, Shen 24A, and Hanfeng A) and their corresponding maintainer lines (Shenchang B, Shen 21B, Shen 22B, Shen 24B, and Hanfeng B) were collected from BT-type CMS seed multiplication fields, whereas seeds of the restorer line Shenhui 26 were obtained from Shenyou 26 hybrid seed production fields. After harvest, seeds were air-dried to a moisture content of approximately 13% prior to storage and subsequent analyses.

To construct the standard curve and evaluate the reliability of the established quantitative detection method, seeds of the BT-type CMS line Shen 21A and its corresponding maintainer line Shen 21B were selected. After dehusking, the rice grains were ground into fine powder using a laboratory high-speed grinder (FW100, Tianjin Taisite Technology Instrument Co., Ltd., Tianjin, China) to ensure sample homogeneity. Rice flour from Shen 21A and Shen 21B was then thoroughly mixed at defined ratios to generate samples with different levels of maintainer-seed contamination.

### 2.2. DNA Extraction

Genomic DNA was extracted from rice flour samples using a commercial DNA extraction kit for edible starch and starch-derived products (RM2301, Biokeystone Technologies Co., Ltd., Chengdu, China) according to the manufacturer’s instructions. Briefly, 100 mg of rice flour was transferred into a 2 mL microcentrifuge tube containing 800 μL of Buffer STS and 20 μL of Proteinase K. The sample was vortexed thoroughly to achieve complete suspension and incubated at 70 °C for 10 min. After centrifugation at 12,000 rpm for 5 min, 300 μL of the supernatant was transferred to a new 2 mL tube. Subsequently, 600 μL of Buffer SWB and 30 μL of starch-binding magnetic beads were added, followed by vortexing for 1 min. The mixture was incubated at room temperature for 10 min, with vortexing for 1 min every 3 min to facilitate DNA binding. The tube was then placed on a magnetic separation rack for approximately 30 s until the beads were completely captured, and the supernatant was carefully removed. The magnetic beads were washed once with 600 μL of Buffer WB and twice with 600 μL of Buffer SPW. After air-drying the beads at room temperature for 5–10 min, DNA was eluted with 50 μL of Buffer TE. The extracted DNA was quantified using a NanoDrop 2000 spectrophotometer (Thermo Scientific, Waltham, MA, USA) and subsequently adjusted to a final concentration of 10 ng/μL for downstream analyses.

### 2.3. Identification of Maintainer-Specific Sequences and Endogenous Reference Genes for Primer Design

Nipponbare, a representative *japonica* cultivar, can serve as a maintainer line for all CMS types currently used in practice, including the BT-type CMS system [15]. To identify maintainer-specific mitochondrial sequences, the mitochondrial genome of Nipponbare (GenBank accession: DQ167400.1) was compared with those of BT-type CMS rice (GenBank accessions: AP017385.1 and AP017386.1) [16,17]. Through this comparative analysis, a Nipponbare-specific mitochondrial sequence present in two copies within the mitochondrial genome was identified. Primers targeting this maintainer-specific sequence were subsequently designed (Forward: TCCCCACTAGTCTTCTTCGTACATG; Reverse: TGGCCCCTAAGAGGAAAAGC). In addition, a conserved mitochondrial sequence present in both CMS and maintainer lines was selected as the endogenous reference target. Primers targeting this reference sequence were designed as follows: Forward, TCGGTATCTATGGACGGCTATG; Reverse, TTGAAGGAAGAAAAGAGGGTGCT. Primer design was performed using Primer Express software 3.0.1 (Thermo Fisher Scientific, Waltham, MA, USA) to minimize potential self-dimer formation, heterodimerization, and secondary structures.

### 2.4. Primer Specificity Analysis by Conventional PCR

To evaluate the specificity of the maintainer-specific primers and the endogenous reference primers, conventional PCR assays were performed using genomic DNA extracted from different rice lines. A no-template control (NTC) was included in each run to monitor potential contamination. PCR amplifications were conducted in a final reaction volume of 20 μL containing 20 ng of genomic DNA, 10 μL of 2× Rapid Taq Master Mix (P222, Vazyme, Nanjing, China), and each primer at a final concentration of 0.25 μM. The PCR cycling conditions were as follows: an initial denaturation at 95 °C for 5 min, followed by 35 cycles of denaturation at 94 °C for 30 s, annealing at 55 °C for 30 s, and extension at 72 °C for 30 s, with a final extension at 72 °C for 5 min. PCR products were resolved by electrophoresis on 2% (*w*/*v*) agarose gels and visualized under UV illumination.

### 2.5. Real-Time PCR Reaction

Quantitative PCR was performed in the LineGene 9600 plus fluorescent quantitative detection system (Bioer Technology, Hangzhou, China). Reactions were carried out in a final volume of 20 μL containing 20 ng of genomic DNA, 10 μL of 2× SYBR qPCR Mix (QPX-201, TOYOBO, Osaka, Japan), and each primer at a final concentration of 0.25 μM. The maintainer-specific primers and the reference primers were amplified in separate reactions. The qPCR cycling conditions were as follows: an initial denaturation at 95 °C for 20 s, followed by 40 cycles of denaturation at 95 °C for 10 s and annealing/extension at 60 °C for 30 s. Each sample was analyzed in three technical replicates.

### 2.6. Construction and Validation of the Standard Curve

Defined seed mixtures were prepared by admixing seeds of the BT-type CMS line Shen 21A with its corresponding maintainer line Shen 21B at proportions of 5%, 2%, 1%, 0.5%, and 0.1%. Four independent experimental batches were generated by two operators on two separate days, with each batch containing all five contamination ratios. Genomic DNA was independently extracted from each mixture, quantified, and normalized. SYBR Green-based real-time PCR was performed with three technical replicates per sample. For each reaction, ΔCt values were calculated as the difference between the Ct value of the maintainer-specific target and that of the reference target (ΔCt = Ct_maintainer_ − Ct_reference_), which was used to normalize for variation in DNA input and amplification efficiency. Mean ΔCt values obtained from the four independent batches were transformed to 2^−ΔCt^ values, which were used to construct a standard curve by linear regression against the corresponding proportions of maintainer-seed contamination. This standard curve was subsequently used to estimate the relative abundance of the maintainer genotype in unknown samples.

### 2.7. Test on Simulated Contamination Samples

To assess the performance of the established qPCR assay for quantifying maintainer-seed contamination, simulated contamination samples were prepared at predefined proportions of 0.20%, 0.60%, 1.06%, and 3.13% by mixing seeds of the BT-type CMS line Shen 21A with its corresponding maintainer line Shen 21B. Genomic DNA was extracted from each simulated sample, quantified, and normalized. SYBR Green-based real-time PCR was performed. Each simulated sample was analyzed in three technical replicates. For each replicate, ΔCt values were calculated as the difference between the Ct value of the maintainer-specific target and that of the reference target. The corresponding maintainer-seed contamination proportions were then estimated by interpolation from the previously constructed standard curve. Recovery was calculated as the ratio of the estimated contamination proportion to the nominal contamination proportion, expressed as a percentage.

## 3. Results

### 3.1. Specificity of the Maintainer-Specific and Reference Primers

**Figure 1:**
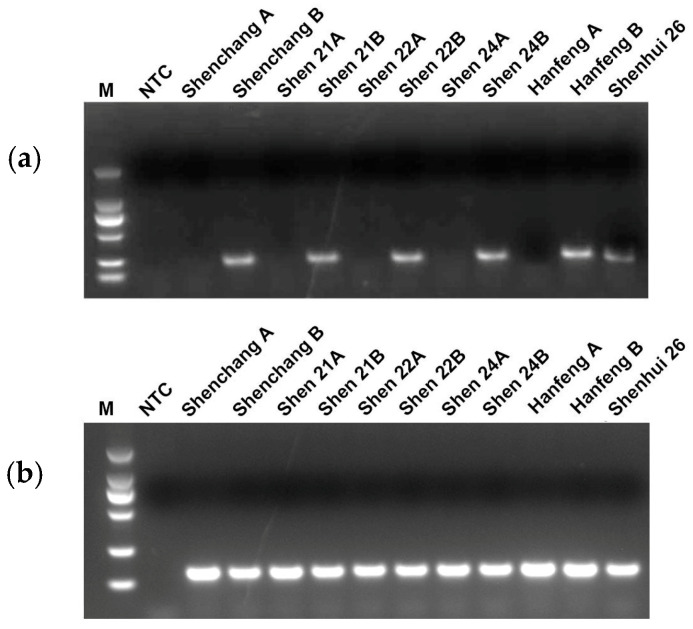
Specificity evaluation of primers by conventional PCR. (**a**) PCR amplification using maintainer-specific primers. (**b**) PCR amplification using primers of the endogenous reference. M, DL2000 DNA marker; NTC, No-template control.

The specificity of the maintainer-specific primers and the endogenous reference primers was first evaluated using conventional PCR. Total genomic DNA extracted from five BT-type CMS lines, their corresponding maintainer lines, and the restorer line Shenhui 26 was used as a template. When the maintainer-specific primers were used, a distinct ~200 bp amplicon was consistently detected in all maintainer lines, whereas no amplification product was observed in any of the CMS lines or in the no-template control (NTC). Notably, the same ~200 bp fragment was also amplified from the restorer line Shenhui 26 (Figure 1a). In contrast, amplification with the reference primers produced a single specific ~130 bp fragment in all tested samples, including CMS lines, maintainer lines, and the restorer line, while no product was detected in the NTC (Figure 1b). These results confirm the high specificity of the maintainer-specific primers and the suitability of endogenous reference primers for subsequent quantitative analyses.

### 3.2. Establishment of a SYBR Green-Based qPCR Assay

**Figure 2:**
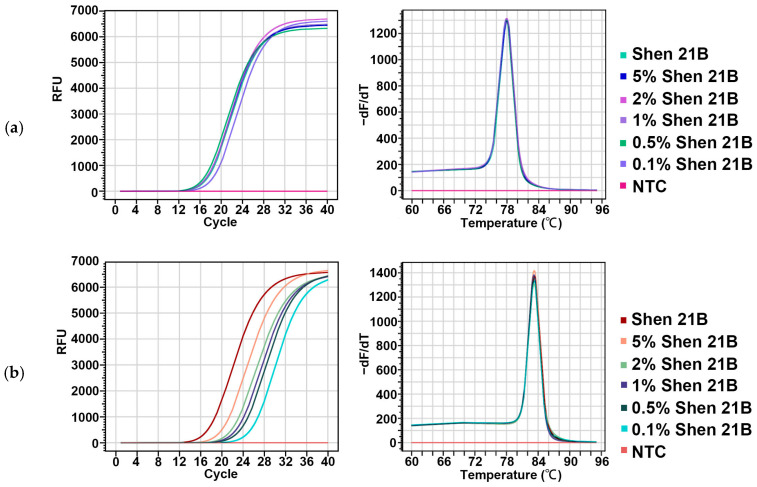
Representative SYBR Green-based real-time PCR amplification and melting curve analysis. (**a**) Amplification curve (**left**) and melting curve (**right**) of the endogenous reference gene. (**b**) Amplification curve (**left**) and melting curve (**right**) of the maintainer-specific target sequence. NTC: no-template control.

To establish a SYBR Green-based quantitative PCR assay for BT-type CMS seed purity detection, seeds of the male-sterile line Shen 21A were admixed with seeds of its corresponding maintainer line Shen 21B at defined proportions (5%, 2%, 1%, 0.5%, and 0.1%). Pure Shen 21B and a no-template control (NTC) were included as positive and negative controls, respectively. Based on the validated primer specificity, real-time PCR was performed separately using the maintainer-specific primers and the endogenous reference primers. When the reference primers were used, all CMS–maintainer mixture samples, as well as the pure maintainer control, generated highly similar amplification curves, and the corresponding Ct values showed minimal variation among samples (Figure 2a), indicating comparable template input and stable amplification of the endogenous reference across samples. In contrast, amplification with the maintainer-specific primers produced clearly distinct amplification profiles among samples with different levels of maintainer-seed contamination. As the proportion of maintainer seeds increased, the Ct values decreased accordingly, reflecting a higher abundance of the maintainer-specific mitochondrial target sequence in the mixed samples (Figure 2b).

Melting curve analysis further confirmed the specificity of the amplification. Both the endogenous reference gene and the maintainer-specific target sequence exhibited single, sharp melting peaks without detectable non-specific amplification or primer–dimer formation (Figure 2a,b). In addition, no amplification signal was observed in the NTC, supporting the absence of contamination.

Together, these results demonstrate that the SYBR Green-based qPCR assay, combining a stable mitochondrial reference with a maintainer-specific target, enables reliable and quantitative discrimination of different levels of maintainer-seed contamination in BT-type CMS seed mixtures.

### 3.3. Construction and Validation of the Standard Curve for CMS Seed Purity Detection

To construct a standard curve for quantitative assessment of CMS seed purity and to evaluate assay reproducibility, seeds of the BT-type CMS line Shen 21A were admixed with seeds of its corresponding maintainer line Shen 21B at defined proportions (5%, 2%, 1%, 0.5%, and 0.1%). Four independent experimental batches were generated by two operators on two separate days, with each batch comprising the full set of five contamination ratios. DNA was extracted independently from each mixture and subjected to SYBR Green-based real-time PCR analysis, with each sample analyzed in three technical replicates.

ΔCt values were calculated for each mixture by subtracting the Ct value of the endogenous reference from that of the maintainer-specific target. As shown in Table 1, ΔCt values exhibited a clear inverse relationship with the proportion of maintainer-seed contamination, with lower ΔCt values observed at higher contamination levels, reflecting the increased abundance of the maintainer-specific mitochondrial sequence. The mean ΔCt values obtained from the four independent DNA batches were subsequently converted to relative abundance using the 2^−ΔCt^ transformation. In all independent sample batches, the reference gene displayed consistent amplification with minimal variation, whereas the maintainer-specific target produced distinct amplification profiles corresponding to the different contamination levels.

**Figure 3:**
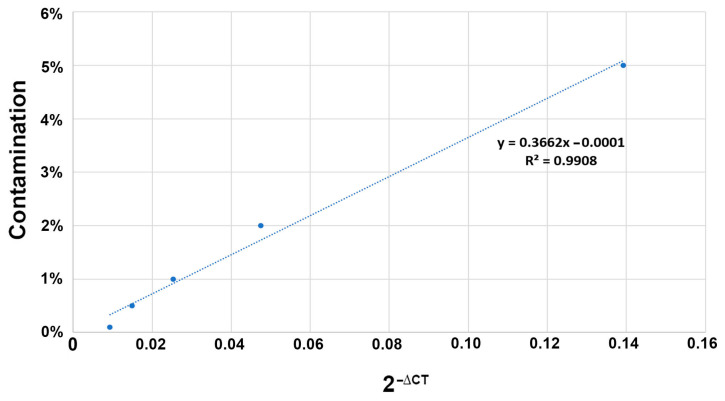
Standard curve generated by plotting the maintainer-seed contamination proportions against the corresponding 2^−ΔCt^ values, where ΔCt was defined as the mean Ct of the maintainer-specific target minus the mean Ct of the endogenous reference gene.

The 2^−ΔCt^ values were plotted against the actual maintainer-seed contamination proportions to generate the standard curve (Figure 3). Linear regression analysis revealed a strong correlation between the two parameters (*R*^2^ > 0.99), demonstrating that the real-time assay established in this study is suitable for the quantification and detection of the genetic purity of BT-type CMS seeds.

The assay showed reliable detection across the tested range (5–0.1%). The lowest contamination level tested that could be consistently detected across independent batches and technical replicates was 0.1%, with stable Ct values observed among replicates. Notably, in practical seed purity assessment, a contamination level exceeding 0.5% is considered unacceptable according to the Chinese national standard (GB 4404.1-2024). Therefore, the sensitivity of the present assay (LOD = 0.1%) is sufficient to reliably detect contamination levels well below the decision threshold, ensuring its suitability for routine screening purposes.

### 3.4. Performance of the qPCR Assay on Simulated Contamination Samples

To evaluate the accuracy of the established qPCR-based approach for quantifying low-level maintainer-seed contamination in BT-type CMS seed lots, simulated contamination samples with predefined proportions (0.20%, 0.60%, 1.06%, and 3.13%) were prepared and analyzed using the previously established standard curve. Each simulated sample was analyzed in three technical replicates.

For each replicate, ΔCt values were calculated by subtracting the Ct value of the endogenous reference target from that of the maintainer-specific target, and the corresponding contamination proportions were interpolated from the standard curve. The calculated contamination levels showed good agreement with the preset values across all tested concentrations (Table 2). Mean recovery values ranged from 87.73% to 95.44%, with relative errors ranging from −12.27% to −4.56%. Although slight underestimation was observed, the deviations remained within an acceptable range for routine seed purity assessment and did not compromise discrimination relative to the regulatory threshold. The variability of replicate measurements was concentration-dependent, with greater dispersion observed at the highest contamination level (3.13%), whereas intermediate and low levels (0.20–1.06%) exhibited comparatively more consistent performance, as reflected by the 95% confidence intervals (CI) (Table 2). These results demonstrate that the established qPCR assay enables reliable quantitative detection of maintainer-seed contamination in BT-type CMS seed lots, including low-level contamination samples.

## 4. Discussion

The Boro II (BT) cytoplasmic male sterility (CMS) system is the first CMS system identified in rice and remains a cornerstone of three-line *japonica* hybrid rice production [14,18]. Maintaining high genetic purity of BT-type CMS seed lots is therefore essential, as even low-level contamination by maintainer seeds can compromise hybrid seed quality and production efficiency [3,6,8,9]. However, conventional purity assessment methods are often time-consuming, labor-intensive, and insufficiently sensitive to detect low proportions of contamination [6]. In this study, we developed a SYBR Green-based qPCR assay that enables rapid, sensitive, and quantitative detection of maintainer-seed contamination in BT-type CMS rice seed lots, providing a rapid molecular approach that may complement conventional seed purity evaluation methods.

The reliability of this assay is largely attributable to its biologically rational target selection and quantitative strategy. CMS is determined by specific mitochondrial genomic features, making mitochondria-derived sequences ideal targets for discrimination between CMS and maintainer lines [11]. The use of a maintainer-specific mitochondrial marker offers a clear advantage in sensitivity due to the high copy number of mitochondrial DNA, particularly when detecting low-level contamination [8,19]. In parallel, the endogenous reference target showed consistent amplification across different seed mixtures (Figure 2a). This stability ensured accurate ΔCt normalization and minimized variability introduced by differences in DNA quantity or quality. Although mitochondrial DNA copy numbers may theoretically vary among different tissues, developmental stages, or genetic backgrounds, the present study was performed using homogenized mature seed samples from closely related CMS and maintainer lines with nearly identical nuclear genetic backgrounds, thereby minimizing potential variation associated with mitochondrial abundance. Under these conditions, the stable amplification of the endogenous mitochondrial reference supported the reliability of the ΔCt-based normalization strategy. By converting ΔCt values to relative abundance using the 2^−ΔCt^ method, the assay achieves reliable quantification without the need for absolute copy number determination, thereby simplifying the workflow and improving operational robustness.

Compared with existing approaches for genetic purity testing, the qPCR-based method described here may offer several practical advantages for routine seed purity assessment. Traditional field-based assessments rely on phenotypic evaluation and are influenced by environmental conditions, developmental stage, and observer experience, which may limit their suitability for rapid or low-level contamination detection [3,6]. Conventional PCR assays primarily provide qualitative results, and analysis of large numbers of seed samples often requires substantial labor for individual DNA extraction, PCR amplification, and gel electrophoresis [6]. Probe-based qPCR systems, such as TaqMan assays, have been reported to improve specificity and reduce background amplification; however, they generally require higher probe design costs and more complex assay optimization. Digital PCR (ddPCR), although capable of absolute quantification and reported to achieve detection limits at single-digit or sub-percent levels in several studies, requires specialized instrumentation and is not yet widely accessible for routine seed testing applications. Capillary electrophoresis-based methods similarly provide high resolution but involve additional post-PCR processing steps, limiting their throughput in large-scale screening. In contrast, the SYBR Green-based strategy employed in this study combines simplicity, cost-effectiveness, and sufficient specificity, suggesting its potential suitability for large-scale routine testing in hybrid rice seed production systems.

In practical hybrid rice seed production, maintainer-line contamination represents the primary concern in BT-type CMS seed purity control because CMS and maintainer lines are cultivated in adjacent multiplication fields. However, according to national seed quality standards (GB 4404.1-2024), all non-CMS contaminants, including maintainer lines and restorer lines, are considered unacceptable in CMS seed lots. In this context, amplification of the maintainer-specific marker in the restorer line Shenhui 26 (Figure 1a) indicates that the present assay does not specifically distinguish maintainer contamination from certain non-CMS genetic backgrounds. Nevertheless, positive amplification signals still provide a practical indicator of compromised CMS seed purity in routine quality-control applications.

The analytical performance of the assay further supports its practical applicability. The strong linearity observed across contamination gradients indicates that the assay operates within a reliable quantitative range suitable for regulatory purity thresholds. Accurate recovery at low contamination levels is particularly relevant for early-stage quality control, where timely intervention can prevent large-scale seed lot rejection. These characteristics collectively suggest that the assay is not merely suitable for laboratory validation but also robust enough for routine implementation in breeding programs and seed production facilities. In the present study, flour-based homogenization was adopted for standard curve establishment to reduce quantitative variation associated with seed-size heterogeneity and improve assay reproducibility during bulk DNA analysis. For practical application in seed production facilities, the present method is intended for analysis of homogenized bulk seed samples rather than individual-seed testing. Prior to DNA extraction, bulked seed samples should be thoroughly homogenized to minimize sampling variation and improve quantitative consistency. The estimated contamination levels obtained by qPCR analysis may then be interpreted relative to the regulatory purity threshold specified in GB 4404.1-2024. In practical quality-control applications, seed lots with estimated contamination levels exceeding the allowable threshold may be considered unsuitable for CMS seed production.

It should also be emphasized that the quantitative strategy established in this study is intended as a production-oriented detection framework rather than a universal assay directly applicable to all BT-type CMS systems using a single calibration model. In the present study, the maintainer-specific primers showed consistent specificity across multiple BT-type CMS/maintainer combinations, including Shenchang A/B, Shen21A/B, Shen22A/B, Shen24A/B, and Hanfeng A/B, indicating that the target sequence is broadly applicable within the tested BT-type CMS systems. However, in practical hybrid rice seed production, different BT-type CMS/maintainer combinations may exhibit variation in mitochondrial sequence composition, seed composition, or amplification characteristics [16,17], thereby requiring system-specific standard curve establishment for accurate quantitative assessment. Accordingly, Shen21A/21B was selected in this study as the representative quantitative validation model because this BT-type CMS system is currently used in our commercial hybrid rice seed production program.

Although the present method demonstrated reliable performance in BT-type CMS rice, several considerations regarding its broader application should be noted. The present method was developed based on BT-type CMS rice and quantitatively validated using the widely cultivated Shen21A/21B system as a representative production model in hybrid rice seed production. For other BT-type CMS/maintainer combinations, recalibration using system-specific standard curves may be required to ensure quantitative accuracy. In addition, application of this strategy to non-BT CMS systems would require identification and validation of corresponding maintainer-specific mitochondrial targets. Nevertheless, the overall framework established here, combining mitochondrial markers, stable endogenous references, and ΔCt-based quantification, provides a flexible strategy that may be adapted to other CMS types and crop species following appropriate target development and calibration. In conclusion, this study provides a rapid, accurate, and cost-effective molecular tool for genetic purity testing of BT-type CMS rice seeds, with practical value for routine quality control in hybrid rice seed production.

## 5. Conclusions

This study developed a SYBR Green-based qPCR assay for rapid and accurate detection of maintainer-seed contamination in BT-type CMS rice seed lots. The assay showed high sensitivity, specificity, and good linearity across a wide range of contamination levels, enabling reliable quantitative assessment. Compared with conventional phenotypic and qualitative PCR methods, it provides a faster, more cost-effective, and high-throughput alternative for routine seed purity testing.

## Figures and Tables

**Table 1:** Inter-operator and inter-batch variation in Ct and ΔCt values in SYBR Green-based qPCR analysis of BT-type CMS seed mixtures.

Contamination Portion	Analyst	Maintainer Specific			Reference			∆Ct	Mean ∆Ct	SD (∆Ct)
		Mean Ct	SD	CV (%)	Mean Ct	SD	CV (%)			
5%	A	21.02	0.01	0.03%	18.08	0.07	0.39%	2.93	2.84	0.15
		20.98	0.01	0.06%	18.01	0.08	0.42%	2.97		
	B	20.88	0.07	0.33%	18.25	0.05	0.26%	2.63		
		20.87	0.05	0.24%	18.02	0.01	0.56%	2.84		
2%	A	22.49	0.15	0.66%	17.95	0.05	0.29%	4.54	4.40	0.10
		22.35	0.04	0.17%	17.97	0.05	0.26%	4.37		
	B	22.45	0.15	0.66%	18.15	0.18	1.00%	4.30		
		22.76	0.13	0.56%	18.39	0.56	3.05%	4.37		
1%	A	23.42	0.11	0.45%	18.12	0.09	0.50%	5.30	5.30	0.02
		23.42	0.06	0.24%	18.09	0.03	0.16%	5.33		
	B	23.35	0.07	0.30%	18.06	0.11	0.63%	5.29		
		23.13	0.08	0.35%	17.84	0.09	0.49%	5.29		
0.5%	A	24.16	0.19	0.80%	18.07	0.08	0.45%	6.09	6.06	0.07
		24.01	0.08	0.31%	18.05	0.04	0.22%	5.96		
	B	24.09	0.21	0.88%	17.95	0.16	0.91%	6.14		
		24.66	0.15	0.60%	18.59	0.33	1.79%	6.07		
0.1%	A	24.48	0.12	0.47%	18.06	0.04	0.21%	6.42	6.75	0.81
		24.87	0.05	0.19%	18.87	0.04	0.19%	6.00		
	B	26.64	0.15	0.55%	18.75	0.17	0.90%	7.89		
		24.57	0.04	0.15%	17.88	0.12	0.66%	6.69		

**Table 2:** Recovery analysis of simulated contamination samples determined by the qPCR assay.

Simulated Sample (%)	Ct Maintainer	Ct Reference	∆Ct	Calculated Contamination (%)	Recovery (%)	Mean Recovery (%)	Mean Error (%)	95% CI (%)
3.13%	21.92	18.48	3.44	3.36% 2.87% 2.73%	107.48% 91.60% 87.24%	95.44%	−4.56%	70.62–120.26
	21.87	18.2	3.67					
	21.87	18.13	3.74					
1.06%	23.38	18.13	5.25	0.95%	89.84%	92.63%	−7.37%	86.05–99.21
	23.31	18.11	5.20	0.99%	93.04%			
	23.22	18.05	5.17	1.01%	95.02%			
0.60%	24.67	18.51	6.16	0.50% 0.54% 0.55%	83.41% 88.88% 92.08%	88.12%	−11.88%	76.72–99.52
	24.54	18.47	6.07					
	24.44	18.42	6.02					
0.20%	26.10	18.40	7.70	0.17%	82.64%	87.73%	−12.27%	74.73–100.73
	26.05	18.43	7.62	0.18%	87.64%			
	25.99	18.45	7.54	0.19%	92.92%			

## Data Availability

The original contributions presented in the study are included in the article; further inquiries can be directed to the corresponding authors.

## Abbreviations

The following abbreviations are used in this manuscript:

BT	Boro II
CMS	Cytoplasmic male sterility
qPCR	Quantitative real-time PCR
GOT	Grow-out-test
NTC	No-template control
LOD	Limit of detection
